# Thermal Inactivation of *Salmonella* in Not‐Ready‐to‐Eat Breaded Stuffed Chicken Products Using an Air Fryer: Impact on Safety, Quality, and Texture

**DOI:** 10.1111/1750-3841.70780

**Published:** 2025-12-17

**Authors:** Ilhami Okur, Gary Sullivan, Jayne Stratton, Byron D. Chaves, Bing Wang

**Affiliations:** ^1^ Department of Food Science and Technology University of Nebraska‐Lincoln Lincoln Nebraska USA; ^2^ Department of Animal Science University of Nebraska‐Lincoln Lincoln Nebraska USA; ^3^ The Food Processing Center University of Nebraska‐Lincoln Lincoln Nebraska USA

**Keywords:** frozen foods, microbial inactivation, poultry meat, thermal lethality, texture

## Abstract

Multiple *Salmonella* outbreaks in the United States have been associated with undercooked not‐ready‐to‐eat (NRTE) breaded stuffed chicken products, which require thorough cooking to ensure pathogen inactivation. Despite manufacturers’ safe handling instructions, increasing consumer use of air fryers for these products poses potential food safety risks when no validated cooking guidelines are currently available. This study evaluated *Salmonella* inactivation and quality changes in NRTE breaded stuffed chicken cooked in two air fryer types (basket and oven‐style) across three temperatures (176.7°C/350°F, 190.6°C/375°F, and 204.4°C/400°F) and five time points (10‐30 min). Results demonstrated that basket‐type air fryers achieved USDA‐FSIS recommended 7‐log *Salmonella* reductions and target internal temperatures (>74°C) faster than oven‐type models: at 25 min (176.7°C/350°F), 20 min (190.6°C/375°F), and 20 min (204.4°C/400°F) versus 30, 30, and 25 min, respectively, for oven‐type units. Color analysis revealed decreasing L* (lightness) and b* (yellowness) values with increasing time/temperature, while a* (redness) values remained higher than control cooking samples. Texture measurements showed MORS force increased with longer processing times. Based on microbiological, color, and texture analysis, specific conditions most closely matched control cooking results: oven‐type air frying at 176.7°C (350°F) for 30 min and basket‐type air frying at 176.7°C (350°F) for 25 min. These findings provide critical data for developing science‐based cooking guidelines for air‐fried NRTE stuffed chicken products, addressing an important food safety gap in consumer practices. Further research should validate these parameters across additional product formulations and air fryer models.

## 1 Introduction

Chicken is one of the major sources of animal protein in the United States, with the per capita consumption increasing from 77.4 lb to 99.6 lb between 2000 and 2023 (NACMCF 2023). However, consumption of contaminated chicken is associated with over 17% of the estimated one million annual cases of salmonellosis in the United States (CDC 2022). Notably, not‐ready‐to‐eat (NRTE) breaded stuffed raw chicken products have been associated with at least 11 outbreaks and approximately 200 illnesses since 1998 (USDA‐FSIS 2022). The USDA‐FSIS conducted a survey to assess the presence of *Salmonella* in NRTE breaded stuffed chicken samples, resulting in 11.48% of the 487 samples collected between July and September 2022 testing positive. Furthermore, the aerobic plate counts (APC) of these samples ranged from 3.69 to 4.05 log CFU/g (USDA‐FSIS 2023).

Due to these concerns, USDA FSIS announced a final determination of declaring *Salmonella* an adulterant in NRTE breaded stuffed raw chicken products when exceeding 1 CFU/g as of May 1, 2024 (USDAFSIS 2024). Despite their pre‐cooked appearance, NRTE breaded stuffed chicken products contain raw chicken and are heat‐treated only to set the batter or breading (USDA FSIS 2022). To ensure microbial safety, consumers must cook these products to a minimum internal temperature of 74°C (165°F) (USDA FSIS 2021).

Air frying is a cooking technique in which hot air circulates uniformly around the food, inducing physical and chemical transformations comparable to those observed in deep frying, but with significantly reduced or no oil absorption (Castro‐López et al. 2023; Fikry et al. 2021). The process operates by heating air at the top of an electric fryer and utilizing a high‐powered fan to distribute heat within a closed chamber rapidly. Food items are exposed directly to the circulating hot air, which promotes surface dehydration and results in the formation of the characteristic crust associated with fried products (Abd Rahman et al. 2017). In recent years, air frying has gained widespread popularity and has been employed for preparing various food items, including chicken nuggets, potatoes, and doughnuts (Cao et al. 2020; Ghaitaranpour et al. 2018; Gouyo et al. 2020). The global air fryer market is projected to reach approximately USD 1.15 billion by 2026 (de Oliveira et al. 2024). In the U.S., approximately 30% of panel participants reported using air fryers to prepare NRTE breaded stuffed chicken (USDA‐FSIS 2022). However, no validated cooking instructions exist for these products in air fryers, raising concerns about their safety and efficacy compared to control cooking methods. Therefore, the objectives of this study were to (1) determine the thermal lethality of *Salmonella* in NRTE breaded stuffed chicken products cooked in an air fryer and (2) assess how their physical and textural characteristics compare with those prepared using the control cooking method.

## 2 Materials and Methods

### 2.1 Sample Preparations and Inoculation

Frozen Cordon Bleu (Barber Foods) were purchased from a local commercial supplier (Lincoln, Neb., USA) and stored at −20°C until analysis. Prior to experimentation, the physical dimensions (length, width, and depth) of 30 individual samples were measured to the nearest millimeter, with results reported as mean values. To evaluate background microflora, APC, coliform, and generic *E. coli* counts were determined in duplicate on 50‐g portions of each sample, 3M APC Petrifilm (Neogen Corp., Lansing, MI, United States) and 3M Coliform/*E. coli* Petrifilm (United States), following the standard protocol outlined in FSIS MLG 3.02, *Quantitative Analysis of Bacteria in Foods as Sanitary Indicators* (USDA FSIS 2015).

A five‐serovar cocktail of *Salmonella enterica* subsp. *enterica* (Enteritidis, Infantis, Kentucky, Schwarzengrund, and Typhimurium) was selected based on USDA‐FSIS prevalence reports of clinically relevant serotypes (Williams et al. 2022). The inoculum was prepared using a modified lawn‐based pelletized method as described by Hildebrandt et al. (2016). Briefly, individual colonies of each strain were separately cultured in tryptic soy broth (Neogen, Corp., Lansing, MI) with 0.6% (w/v) yeast extract (TSBYE) at 37°C for 24 h through two sequential incubations: the first initiated from the frozen stock culture (10 mL TSBYE, 37°C, 24 h), and the second prepared by transferring 1 mL of the first culture into 9 mL of fresh TSBYE and incubating under the same conditions. For lawn preparation, 1 mL of each overnight culture was spread‐plated onto tryptic soy agar (Neogen, Inc., Lansing, MI) with 0.6% yeast extract (TSAYE) and incubated (24 ± 2 h, 37°C). Lawns were harvested using 10 mL of 0.1% peptone water (PW; Fisher Scientific, Waltham, MA, USA) and dislodged with a sterile L‐shaped spreader. Equal volumes of each strain suspension were combined, centrifuged (3,000 *g*, 15 min), and the pellet was resuspended in 50 mL of 0.1% PW. The final inoculum concentration was estimated at 10.2 ± 0.3 log CFU/mL.

NRTE breaded stuffed chicken samples were thawed at 4°C. Samples were injected with 0.50 mL of the *Salmonella* cocktail with a 1 mL syringe, resulting in a final concentration of 8.2 ± 0.1 log CFU/g. Following inoculation, the samples were stored at −20°C for a minimum of 24 h to allow the samples to freeze. The product was expected to be cooked from frozen, and that was why it was frozen immediately after inoculation.

### 2.2 Thermal Treatment of NRTE Breaded Stuffed Chicken Samples

Two air fryer models were used for thermal inactivation: Ninja AF101 (basket‐type) and Ninja SP101 (oven‐type), selected based on their status as top‐selling models in their respective categories. Samples were cooked at 176.7°C (350°F), 190.6°C (375°F), and 204.4°C (400°F) for 10, 15, 20, 25, or 30 min, with untreated samples serving as controls. Each treatment was performed in triplicate, with successive runs initiated only after the air fryer returned to room temperature. As a control cooking method, NRTE breaded stuffed chicken samples were prepared using the baking function of the oven‐type air fryer (Ninja SP101). Specifically, samples were cooked at 204.4°C (400°F) for 34 min, following the manufacturer's recommended time and temperature settings for optimal food safety and quality. Real‐time temperature monitoring was conducted using Type T thermocouples (Omega, Norwalk, CT, USA) positioned at the center of the bottom of the basket or oven, with data recorded at 1‐min intervals. Additionally, the internal temperature of each NRTE breaded stuffed chicken sample was measured immediately post‐treatment.

### 2.3 Microbiological Analysis

After thermal treatment, samples were kept at room temperature until their surface temperature reached equilibrium with the surrounding environment. To enumerate *Salmonella* survivors, 50‐g samples were aseptically transferred to sterile plastic bags and diluted 1:10 in 0.1% peptone water. Appropriate serial dilutions were prepared and plated in duplicate on modified tryptic soy agar (mTSA) containing 0.6% (w/v) yeast extract, ferric citrate (0.05%; Sigma‐Aldrich, St. Louis, MO), and sodium thiosulfate (0.03%; Sigma‐Aldrich). After 24 h of incubation at 37°C, black‐centered colonies were counted as *Salmonella*. Results were converted to log CFU/g. The limit of detection (LOD) was lower than 10 CFU/g. For samples with counts below the LOD, samples were first enriched in buffered peptone water (BPW; Sigma‐Aldrich, St. Louis, MO, USA) and incubated at 37°C for 18–24 h. The enrichment cultures were then streaked onto XLD agar (Neogen Corp., Lansing, MI, USA) and incubated at 37°C for 18–24 h. Plates exhibiting typical *Salmonella* colonies, characterized by black centers with an opaque zone, were recorded as *Salmonella*‐positive.

### 2.4 Color and Texture Analysis

Only air fryer conditions demonstrating validated microbial safety were selected for quality evaluation. The safety threshold was established based on (1) achieving a minimum 7‐log CFU/g reduction of *Salmonella*, consistent with USDA‐FSIS standards (FSIS 2021), and (2) attaining a core temperature ≥74°C (165°F), the USDA‐recommended safe endpoint temperature for poultry (USDA‐FSIS 2021).

Color analysis was conducted using a handheld colorimeter (CR‐300, Konica Minolta, Ramsey, NJ, USA). L*, a*, and b* values were recorded from three different surface locations on each sample cooked in the air fryers and the control cooking method. The color difference (ΔE) was determined using the following equation:

(1) $$\Delta E=\sqrt{{\left(L^{*}-L_{0}^{*}\right)}^{2}+{\left(a^{*}-a_{0}^{*}\right)}^{2}+{\left(b^{*}-b_{0}^{*}\right)}^{2}}\;$$

where L_0_*, a_0_*, and b_0_* respectively represented lightness, redness, and yellowness.

A texture analysis was performed to evaluate the shear force (N) using the Meullenet‐Owens Razor Shear (MORS) method. Each sample was tested using a Texture Analyzer (Model TMS‐PRO, Food Technology Corp., Sterling, VA, USA) equipped with a 50‐kg load cell. The shear distance was at least 2 cm, with a blade test speed of 10 mm/s and a penetration depth of 40 mm. Three shear measurements were conducted per sample. All analyses were performed in triplicate.

### 2.5 Statistical Analysis

Statistical analyses were conducted using Minitab software (version 16.0). Analysis of variance (ANOVA) was employed to assess the significance of the independent variables. Tukey's multiple comparison test was applied to determine significant differences among the experimental mean values. Differences were considered statistically significant at *P* ≤ 0.05.

## 3 Results

### 3.1 Sample Characterization

The average dimension of the samples was 113.5 ± 2.5 mm by 61.9 ± 2.2 mm, 40 ± 1.1 mm in depth, and the average weight of the samples were 198 g (7 oz). Total APC of samples were 3.47 ± 0.07 log CFU/g. The coliform count and *Enterobacteriaceae* of the samples were below LOQ (<2.2 log CFU/g).

### 3.2 Thermal Performance of Air Fryers

The thermal profiles of oven‐type and basket‐type air fryers were evaluated during cooking of NRTE breaded stuffed chicken, which had an initial core temperature of ‐16°C. As shown in Table 1, both appliance types successfully achieved the USDA‐FSIS recommended safe endpoint internal temperature of 74°C (165°F), though with notable differences in time efficiency. The oven‐type air fryer required 30 min at both 176.7°C (350°F) and 190.6°C (375°F), and 25 min at 204.4°C (400°F) to reach the target temperature. In contrast, the basket‐type air fryer demonstrated faster heating, achieving the safe temperature in 25 min at 176.7°C (350°F) and just 20 min at higher temperature settings (190.6°C/375°F and 204.4°C/40 0°F), representing a 25%–50% reduction in cooking time compared to the oven‐type model.

**TABLE 1:** Actual geometric center temperature of breaded stuffed chicken samples during cooking in two air frying appliances set to various cooking temperatures (*n* = 3).

Time (min)	Basket‐type			Oven‐type		
	176.7°C (350°F)	190.6°C (375°F)	204.4°C (400°F)	176.7°C (350°F)	190.6°C (375°F)	204.4°C (400°F)
10	3.2 ± 3.0^c^	2.65 ± 0.2^d^	5.7 ± 4.7^b^	5.3 ± 2.1^d^	7.2 ± 0.9^e^	9.7 ± 0.4^d^
15	16.5 ± 9.5^c^	35.9 ± 1.8^c^	34.6 ± 10.4^b^	11.5 ± 5.7^d^	19.8 ± 2.0^d^	25.2 ± 3.8^c^
20	57.3 ± 4.9^b^	75.7 ± 5.2^b^	79 ± 1.7^a^	33.0 ± 1.0^c^	36.3 ± 0.8^c^	49.5 ± 1.0^b^
25	84.8 ± 2.6^a,b^	93.2 ± 1.2^a^	93.6 ± 9.5^a^	56.6 ± 1.3^b^	67.2 ± 1.3^b^	84.7 ± 3.6^a^
30	88.0 ± 1.9^a^	95.7 ± 1.8^a^	99.9 ± 6^a^	76.5 ± 1.1^a^	91.7 ± 0.8^a^	92.2 ± 1.0^a^

*Note*: Values represented with different superscript letters are statistically different at *p*  <  0.05.

Temperature measurements revealed operational variations between setpoints and actual performance. While both units were programmed for identical temperature ranges (176.7‐204.4°C/350‐400°F), the oven‐type air fryer consistently operated below its nominal settings, reaching maximum recorded temperatures of 161°C, 180°C, and 187°C for the respective temperature settings. The basket‐type unit more closely matched its programmed temperatures, achieving 167°C, 177°C, and 205°C, with the highest setting slightly exceeding its designated temperature (Figure S2).

**FIGURE 1:**
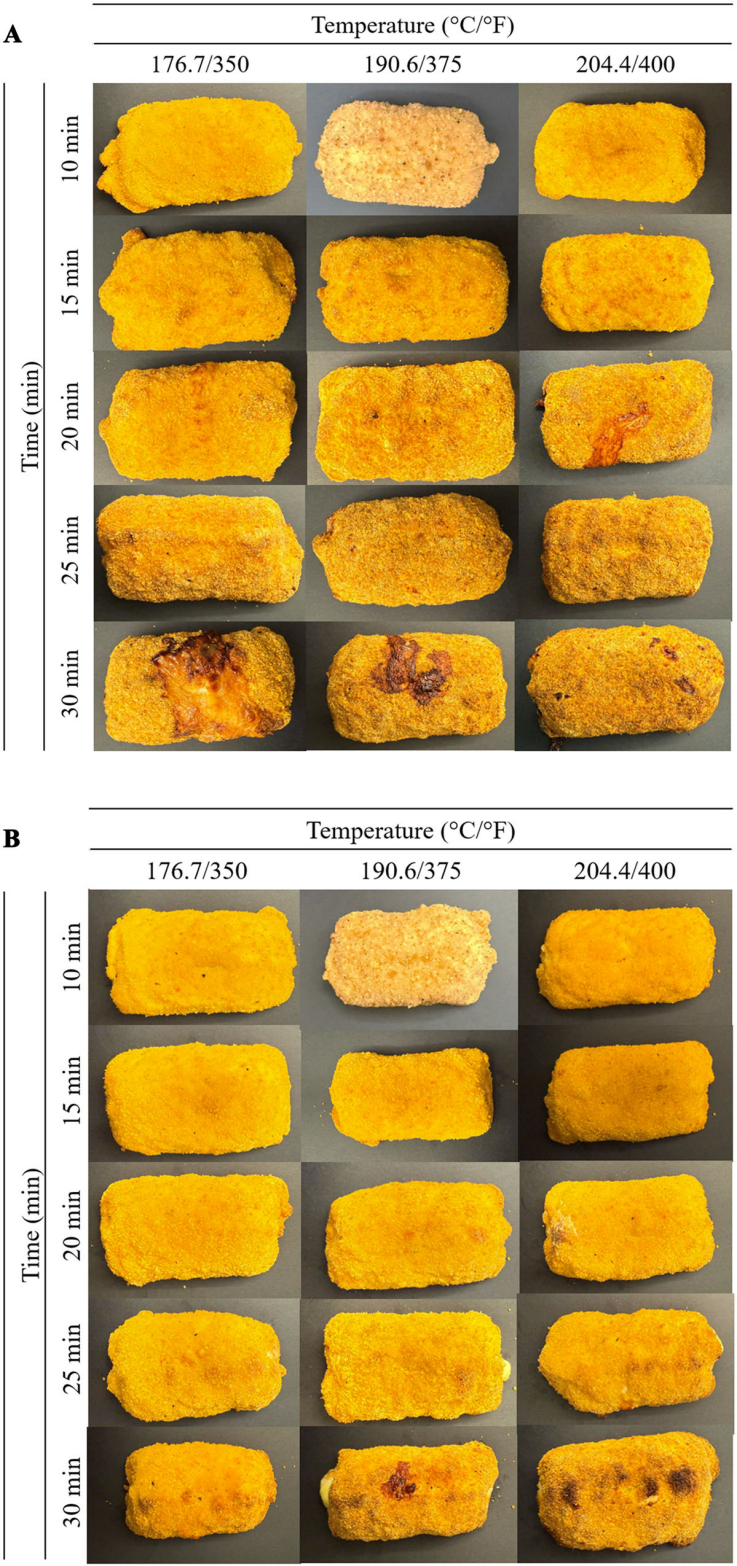
(A) Top view of NRTE breaded stuffed chicken samples treated with basket‐type air fryer treatments. (B) Top view of NRTE breaded stuffed chicken samples treated with oven‐type air fryer treatments.

Comparative analysis of cooked products (Figures 1 and S1) demonstrated that the oven‐type air fryer produced more uniform browning across the chicken surfaces compared to the basket‐type unit. This enhanced browning pattern likely resulted from the oven‐type's more gradual temperature increase and more uniform heat distribution characteristics, which may promote even crust formation during cooking.

### 3.3 *Salmonella* Inactivation in NRTE Breaded Stuffed Chicken

Both air fryer types achieved complete *Salmonella* reduction to below the detection limit (1 log CFU/g) in NRTE breaded stuffed chicken samples (Table 2). The basket‐type air fryer demonstrated superior inactivation efficiency, achieving a critical 7‐log reduction at 176.7°C (350°F) in 25 min and at higher temperatures (190.6°C/375°F and 204.4°C/400°F) in 20 min. In comparison, the oven‐type unit required longer exposure times: 30 min at both 176.7°C (350°F) and 190.6°C (375°F), and 25 min at 204.4°C (400°F) to attain equivalent reduction.

**TABLE 2:** *Salmonella* logarithmic reduction in NRTE breaded stuffed chicken samples treated with different air fryers (*n* = 3 per treatment condition).

Temperature (°C, °F)	Time (min)	Log Reduction (log CFU/g)	
		Basket‐type	Oven‐type
176.7 (350)	10	0.20 ± 0.07^d^	0.08 ± 0.03^f^
	15	0.95 ± 0.40^b,c,d^	0.07 ± 0.04^f^
	20	3.99 ± 0.22^a^	1.64 ± 0.12^e^
	25	>7.2^*^	3.47 ± 0.34^c^
	30	>7.2	>7.2^*^
190.6 (375)	10	0.53 ±0.15^c,d^	0.04 ± 0.02^f^
	15	1.00 ± 0.18^b,c^	0.19 ± 0.07^f^
	20	>7.2^*^	3.10 ± 0.09^d^
	25	>7.2^*^	4.79 ± 0.07^a^
	30	>7.2	>7.2^*^
204.4 (400)	10	0.51 ± 0.47^b,c,d^	0.10 ± 0.07^f^
	15	1.34 ± 0.49^b^	0.15 ± 0.08^f^
	20	>7.2^*^	3.98 ± 0.13^b^
	25	>7.2^*^	>7.2^*^
	30	>7.2	>7.2

* Samples tested positive for *Salmonella* after enrichment, indicating residual viability below the direct detection limit.

Values represented with different superscript letters are statistically different at *p  < 0 .05*.

The most significant microbial reduction occurred when samples reached the critical internal temperature of 74°C (165°F), which corresponded to 15–25 min in basket‐type units versus 20–30 min in oven‐type models. Post‐enrichment analysis revealed residual *Salmonella* viability below detection limits in some samples: oven‐type treatments at 176.7°C (350°F) and 190.6°C (375°F) for 30 min, and 204.4°C (400°F) for 25 min showed positive enrichment results, while extended 30‐min treatment at 204.4°C (400°F) yielded negative results. Similarly, basket‐type samples remained enrichment‐positive after 25 min at all temperatures but achieved complete inactivation (enrichment‐negative status) after 30 min of treatment.

### 3.4 Color Analysis of Cooked Products

Analysis revealed that both air fryer types showed decreasing L* and b* values with increasing treatment temperature and duration, indicating progressive darkening and reduced yellowness. Notably, air‐fried samples consistently exhibited higher a* values (enhanced redness) compared to control products, though no clear temperature‐ or time‐dependent trends emerged for this parameter.

The total color change (ΔE) analysis demonstrated that specific conditions most closely matched control cooking results: oven‐type air frying at 176.7°C (350°F) for 30 min and basket‐type air frying at 176.7°C (350°F) for 25 min (Table 3).

**TABLE 3:** Surface color of NRTE breaded stuffed chicken samples treated with (A) basket‐type air fryer and (B) oven‐type air fryer.

Parameter (°C/°F‐min)	L*	a*	b*	ΔE
A				
Control	59.5 ± 1.1^a^	9.1 ± 0.6^d^	50.5 ± 1.3^a^	—
176.7/350‐25	50.3 ± 1.7^b^	11.2 ± 0.5^b,c,d^	37.4 ± 0.4^b^	16.2
176.7/350‐30	50.0 ± 0.9^b^	11.6 ± 0.8^a,b,c^	34.6 ± 1.4^c,d^	18.8
190.6/375‐20	45.3 ± 0.6^c^	13.4 ± 0.5^a,b^	36.3 ± 0.5^b,c^	20.5
190.6/375‐25	43.4 ± 1.8^c^	12.0 ± 0.7^a,b,c^	32.7 ± 0.1^d,e^	24.2
190.6/375‐30	43.7 ± 1.1^c^	10.5 ± 0.7^c,d^	32.6 ± 1.5^d,e^	24
204.4/400‐20	45.0 ± 0.3^c^	13.7 ± 1.3^a^	36.4 ± 0.3^b,c^	20.8
204.4/400‐25	42.2 ±0.9^c^	13.4 ± 1.0^a,b^	33.5 ± 1.2^d,e^	24.6
204.4/400‐30	38.3 ±1.0^d^	12.8 ± 0.3^a,b^	31.0 ± 0.6^e^	29.1
B				
Control	59.5 ± 1.1^a^	9.1 ± 0.6^b^	50.5 ± 1.3^a^	—
176.7/350‐30	53.1 ± 1.4^a,b^	11.3 ± 1.2^a,b^	48.6 ± 1.0^a^	6.5
190.6/375‐30	49.3 ± 2.09^b,c^	11.6 ± 1.0^a,b^	37.7 ± 2.0^c^	16.6
204.4/400‐25	53.5 ± 0.3^a,b^	11.6 ± 0.7^a,b^	44.2 ± 1.2^b^	9.1
204.4/400‐30	45.7 ± 4.6^c^	13.0 ± 1.32^b^	34.8 ± 0.8^c^	21.2

*Note*: Values represented with different superscript letters are statistically different at *p*  <  0.05.

### 3.5 Texture Analysis Using Meullenet‐Owens Razor Shear (MORS) Force

The shear properties of air‐fried samples, presented in Table 4, revealed distinct textural characteristics compared to control cooking preparation (reference curve in Figure S3). Analysis of the shear curves identified two characteristic peaks corresponding to (1) the breaded coating/chicken layer and (2) the ham component, with cheese melting during cooking creating this discernible separation.

**TABLE 4:** MORS results of NRTE breaded stuffed chicken samples treated with (A) basket‐type air fryer and (B) oven‐type air fryer.

Parameter (°C/°F‐min)	Peak 1	Peak 2
A		
Control	0.51 ± 0.04^e^	1.39 ± 0.09^a^
176.7/350‐25	0.60 ± 0.06^d,e^	1.44 ± 0.21^a^
176.7/350‐30	0.68 ± 0.09^c,d,e^	1.39 ± 0.02^a^
190.6/375‐20	0.85 ± 0.10^b,c,d^	1.36 ± 0.27^a^
190.6/375‐25	0.92 ± 0.20^a,b,c^	1.36 ± 0.20^a^
190.6/375‐30	1.17 ± 0.10^a,b,c^	1.34 ± 0.05^a^
204.4/400‐20	0.94 ± 0.12^a,b^	1.48 ± 0.06^a^
204.4/400‐25	1.02 ± 0.13^a,b^	1.43 ± 0.06^a^
204.4/400‐30	1.16 ± 0.08^a^	1.53 ± 0.06^a^
B		
Control	0.51 ± 0.04^b^	1.39 ± 0.09^a^
176.7/350‐30	0.61 ± 0.19^a,b^	1.48 ± 0.03^a^
190.6/375‐30	0.68 ± 0.10^a,b^	1.33 ± 0.11^a^
204.4/400‐25	0.70 ± 0.05^a,b^	1.41 ± 0.12^a^
204.4/400‐30	0.81 ±0.03^a^	1.35 ± 0.10^a^

*Note*: Values represented with different superscript letters are statistically different at *p* < 0.05.

Key findings demonstrated significant differences in first‐peak shear force values (*P* < 0.05), where control cooking samples showed the lowest resistance (0.51 ± 0.04 kgf), while basket‐type air frying at 204.4°C (400°F) for 30 min produced the firmest texture (1.16 ± 0.08 kgf). However, the second‐peak analysis revealed no statistically significant differences between cooking methods (*P* > 0.05).

## 4 Discussion

The growing consumer adoption of air fryers for preparing NRTE breaded stuffed chicken products highlights a critical gap in food safety guidance, as no validated cooking parameters currently exist for these appliances/food combinations. This study provides the first comprehensive evaluation of *Salmonella* inactivation in NRTE products across different air fryer types (basket vs. oven), temperatures, and cooking times. The key finding that basket‐type units achieve faster thermal processing than oven‐type models (reaching 88°C vs. 76.5°C at 176.7°C/350°F for 30 min) can be attributed to fundamental design differences. The air fryer temperature profile also supports the design differences (Figure S2). As described by Lee and Neio Demirci (2022), the basket‐type's compact cooking chamber and integrated door system create a more efficient heat transfer environment. The smaller air volume requires less energy for preheating and maintains higher convective heat flux during operation, explaining both the faster cooking times and higher endpoint temperatures observed in this study. These design advantages may have significant implications for both food safety outcomes and energy efficiency in household cooking.

These findings demonstrate that both air fryer types can achieve the USDA FSIS recommended 7‐log *Salmonella* reduction (USDA FSIS 2021), though with notable time differences. The oven‐type air fryer required 25–30 min depending on temperature (176.7‐204.4°C), while the basket‐type achieved equivalent reduction in 20–25 min—consistent with its superior heating efficiency. All effective treatments exceeded the 74°C internal temperature threshold, validating their safety efficacy.

These results contrast with previous reports of shorter processing times for thinner poultry products. Rao et al. (2020) observed 74°C attainment in just 8.5 min for 12‐mm chicken strips (‐10°C initial temperature), while Cano et al. (2022) reported 7‐log reduction in ≤8 min for chicken wings. The substantially longer times required in this study (40‐mm thick samples) strongly support the critical role of product geometry in thermal processing. As Shen et al. (2010) established, increased thickness significantly delays heat penetration to the geometric center, necessitating extended processing for equivalent pathogen reduction. This thickness‐dependent effect explains why this stuffed chicken products required nearly 3 times longer cooking times than thinner cuts to achieve equivalent *Salmonella* inactivation, despite similar air frying temperatures. The practical implication is clear: validated cooking instructions must account for product‐specific geometry rather than assuming universal parameters. This data provides crucial benchmarks for developing science‐based guidelines for thicker NRTE stuffed poultry products in home cooking applications.

Color represents a fundamental quality attribute of fried foods, significantly influencing consumer perception and acceptability (Aliberti et al. 2025; Manjunatha et al. 2014; Téllez‐Morales and Arce‐Ortiz 2024). These results demonstrated consistent decreases in L* (lightness) and b* (yellowness) values with increasing processing time and temperature for both air fryer types, while a* (redness) values showed inconsistent patterns. Previous studies report similar color transformations under comparable processing conditions. Aliberti et al. (2025) observed L* value reductions from 49.9 to 44.9 and b* decreases from 45.6 to 39.7 in chicken nuggets processed in basket‐type air fryers (180‐200°C, 10–20 min). Comparable trends emerged in oven‐type units, where extended processing (200°C, from 20 to 25 min) decreased L* values from 49.1 to 43.3 (Aliberti et al. 2025). Castro‐López et al. (2023) further confirmed these patterns, demonstrating temperature‐dependent L* reductions from 50 to 43.17 (190°C) and 45.39 (160°C) in chicken nuggets, with parallel b* value decreases during processing.

This phenomenon extends beyond poultry products, as evidenced by studies on fish skin, frozen batter‐coated foods, and plant‐based fishballs all reporting progressive L* value decreases with extended thermal processing (Bhuiyan and Ngadi 2024; Fang et al. 2021; Ran et al. 2023). The consistent darkening across diverse food matrices suggests fundamental physicochemical changes—likely Maillard reaction progression and surface dehydration—inherent to air frying technology.

Texture represents a critical quality parameter in fried foods, particularly the development of a desirable crispy crust that consumers prefer. These textural changes occur through complex physicochemical transformations, including water evaporation, protein denaturation, starch gelatinization, and crust formation (Asokapandian et al. 2020; Cao et al. 2020; Teruel et al. 2015). This study demonstrated that increased frying time and temperature consistently increased the MORS force values for NRTE breaded stuffed chicken in both air fryer types, indicating progressive texture firming.

This trend aligns with established literature on air‐fried products. Aliberti et al. (2025) reported increasing hardness in chicken nuggets from 16 to 21 N (180°C, from 10 to 15 min) and 23 to 30 N (190°C, from 15 to 20 min) using basket‐type air fryers, with comparable hardening observed in oven‐type units (from 17 to 22 N at 200°C, from 20 to 25 min). Similarly, Cao et al. (2020) documented substantial texture firming in chicken nuggets during extended processing (from 0 to 18 min at 180°C). Castro‐López et al. (2023) further validated these findings, showing penetration force increases from 8.5 to 35 kg across various temperature‐time combinations (160–190°C, 0–15 min), attributing these changes to moisture loss and crust development.

Similar to the color development, this textural transformation phenomenon extends beyond poultry products, having been observed in diverse food matrices including Clearhead Icefish and plant‐based fishballs (Li et al. 2025; Ran et al. 2023), suggesting fundamental principles of air frying texture modification applicable across food categories.

In the literature, many studies have examined both the thermal intensity (time–temperature combinations) required to inactivate *Salmonella* and how these heat treatments affect product quality attributes such as texture, color, and sensory characteristics. The consensus across studies is that there is a trade‐off: higher thermal intensity (higher temperature and/or longer time) increases *Salmonella* log reductions but might degrade texture, color, and other sensory properties. However, safety targets can be achieved while maintaining acceptable quality through appropriate process design. Based on microbiological, color, and texture analyses, the specific conditions that most closely matched the control cooking results in this study were oven‐type air frying at 176.7°C (350°F) for 30 min and basket‐type air frying at 176.7°C (350°F) for 25 min. Further extension of cooking time at these temperatures resulted in excessive surface browning and darker color, indicating overprocessing (Figures 1 and S1). These findings indicate that the safety‐assuring conditions identified in this study might simultaneously guarantee desirable product quality. Qu et al. (2021) investigated the effects of different cooking conditions (60–90°C, 0–65 min) on microbial and quality changes in frozen and fresh chicken livers. Cooking to an internal temperature of 70–73.9°C followed by a holding time of 10–126 s was recommended for food processing plants and restaurants preparing ready‐to‐eat meals containing chicken livers, as these conditions ensured microbial safety against *Salmonella* while preserving the desired texture and pink color. Further extension of cooking time at these temperatures resulted in a denser, coarser texture in the cooked livers.

## 5 Conclusion

This study evaluated the thermal inactivation of *Salmonella* and quality changes in NRTE breaded stuffed chicken products prepared using two different air fryer types. The results of this study demonstrate that both basket‐type and oven‐type air fryers can achieve the USDA‐FSIS recommended 7‐log reduction of *Salmonella*, though with distinct time‐temperature profiles. The basket‐type air fryer showed superior thermal transfer efficiency, reaching target inactivation 25%–50% faster than the oven‐type model, likely due to its more compact cooking chamber and efficient heat distribution. All effective treatments exceeded the critical internal temperature of 74°C while maintaining acceptable product quality, as evidenced by controlled color development and desirable texture formation. It is important to note that some intermediate temperatures resulted in cellular injury rather than complete inactivation of *Salmonella*, as indicated by the detection of surviving cells after enrichment in certain samples. This highlights the critical importance of achieving the recommended internal temperature to achieve a higher level of microbial safety. The extended processing times required for these thicker stuffed products (40 mm) compared to previously reported values for thinner poultry items highlight the crucial role of product geometry in thermal processing. This thickness‐dependent effect was particularly evident in texture development, where increased processing times produced firmer products through enhanced crust formation. Color parameters followed expected trends of decreasing lightness and yellowness with extended processing, consistent with Maillard reaction progression observed in other air‐fried products. These findings provide essential scientific data to support the development of cooking guidelines for NRTE stuffed poultry products in air fryers, addressing a significant gap in current food safety recommendations. The established time‐temperature combinations offer practical benchmarks for consumers while maintaining product quality attributes. Future studies should explore the validation of these parameters across different product formulations and air fryer models to further strengthen food safety practices for this increasingly popular cooking method.

## Author Contributions

**Ilhami Okur**: conceptualization, methodology, data curation, formal analysis, visualization, writing – original draft, writing – review and editing, validation, software. **Gary Sullivan**: methodology, writing – review and editing, resources. **Jayne Stratton**: methodology, writing – review and editing. **Byron D. Chaves**: writing – review and editing. **Bing Wang**: conceptualization, methodology, investigation, formal analysis, supervision, funding acquisition, resources, project administration, writing – review and editing, writing – original draft.

## Conflicts of Interest

The authors declare no conflicts of interest.

## Supporting information




**Supplementary Materials**: jfds70780‐Sup‐0001‐SuppMat.docx
