# Supplementary material for: Thermal Inactivation of Salmonella in Not‐Ready‐to‐Eat Breaded Stuffed Chicken Products Using an Air Fryer: Impact on Safety, Quality, and Texture
Source: J Food Sci. 2025 Dec 17;90(12):e70780. doi: 10.1111/1750-3841.70780 (PMC12712419; doi:10.1111/1750-3841.70780)
Supplement: Supplementary file 1 — Supplementary Materials: jfds70780‐Sup‐0001‐SuppMat.docx [file JFDS-90-0-s001.docx]

**Thermal Inactivation of *Salmonella* in** **Not-Ready-to-Eat Breaded Stuffed Chicken Products Using an Air Fryer: Impact on Safety, Quality and Texture**

Ilhami Okur^1^, Gary Sullivan^2^, Jayne Stratton^1,3^, Byron D. Chaves^1^, Bing Wang^1*^

^1^ Department of Food Science and Technology, University of Nebraska-Lincoln, Lincoln, NE 68588, United States

^2^ Department of Animal Science, University of Nebraska-Lincoln, Lincoln, NE 68583, United States

^3^ The Food Processing Center, University of Nebraska-Lincoln, NE 68588, USA

* Corresponding author

Department of Food Science and Technology, University of Nebraska-Lincoln, Lincoln, NE 68588

[bing.wang@unl.edu](mailto:bing.wang@unl.edu)

**Figure S1A:** Side view of Cordon Bleu treated with different basket-type air fryer treatments

**
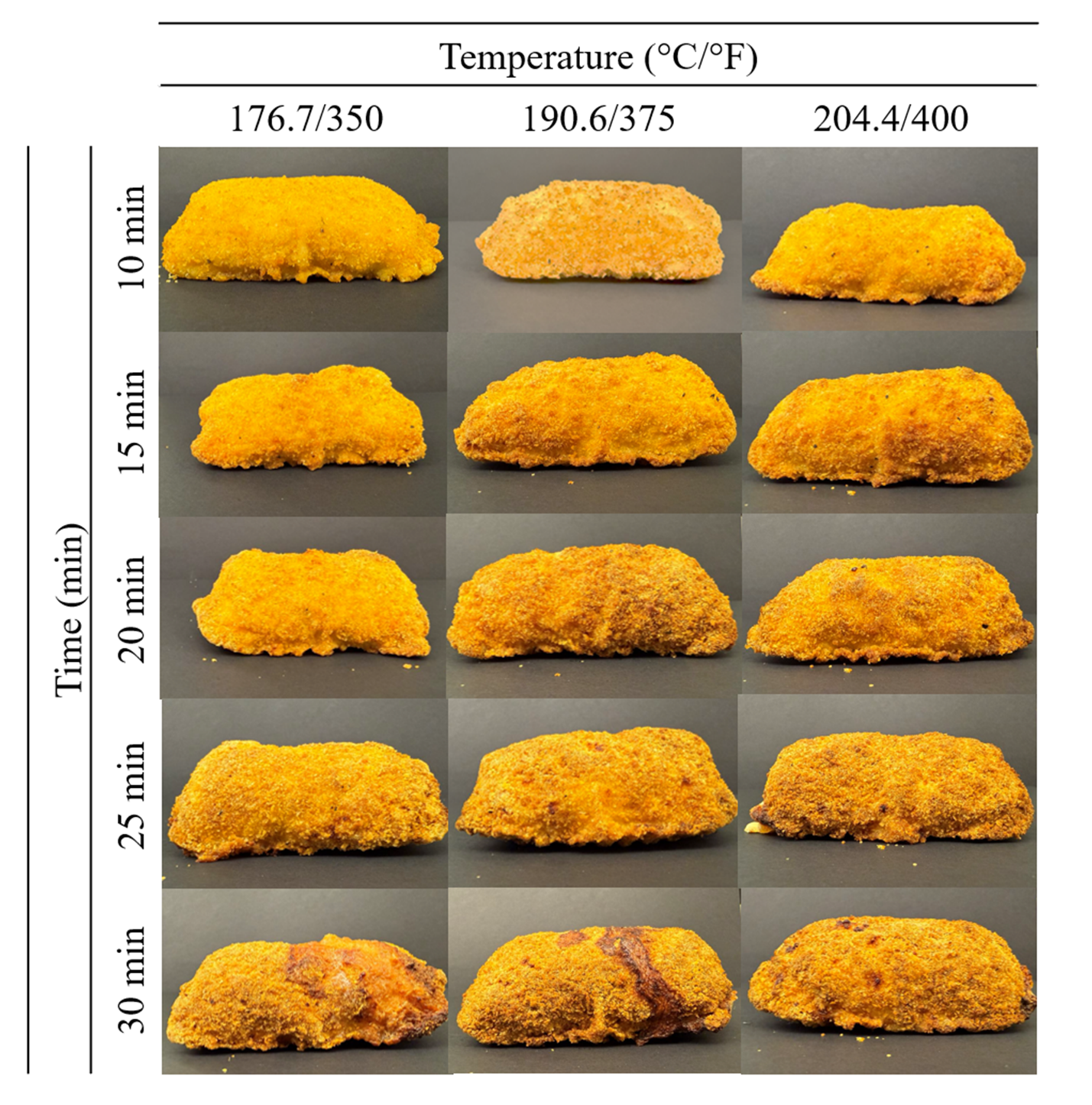
**

**Figure S1B.** Side view of Cordon Bleu treated with different basket-type air fryer treatments

**
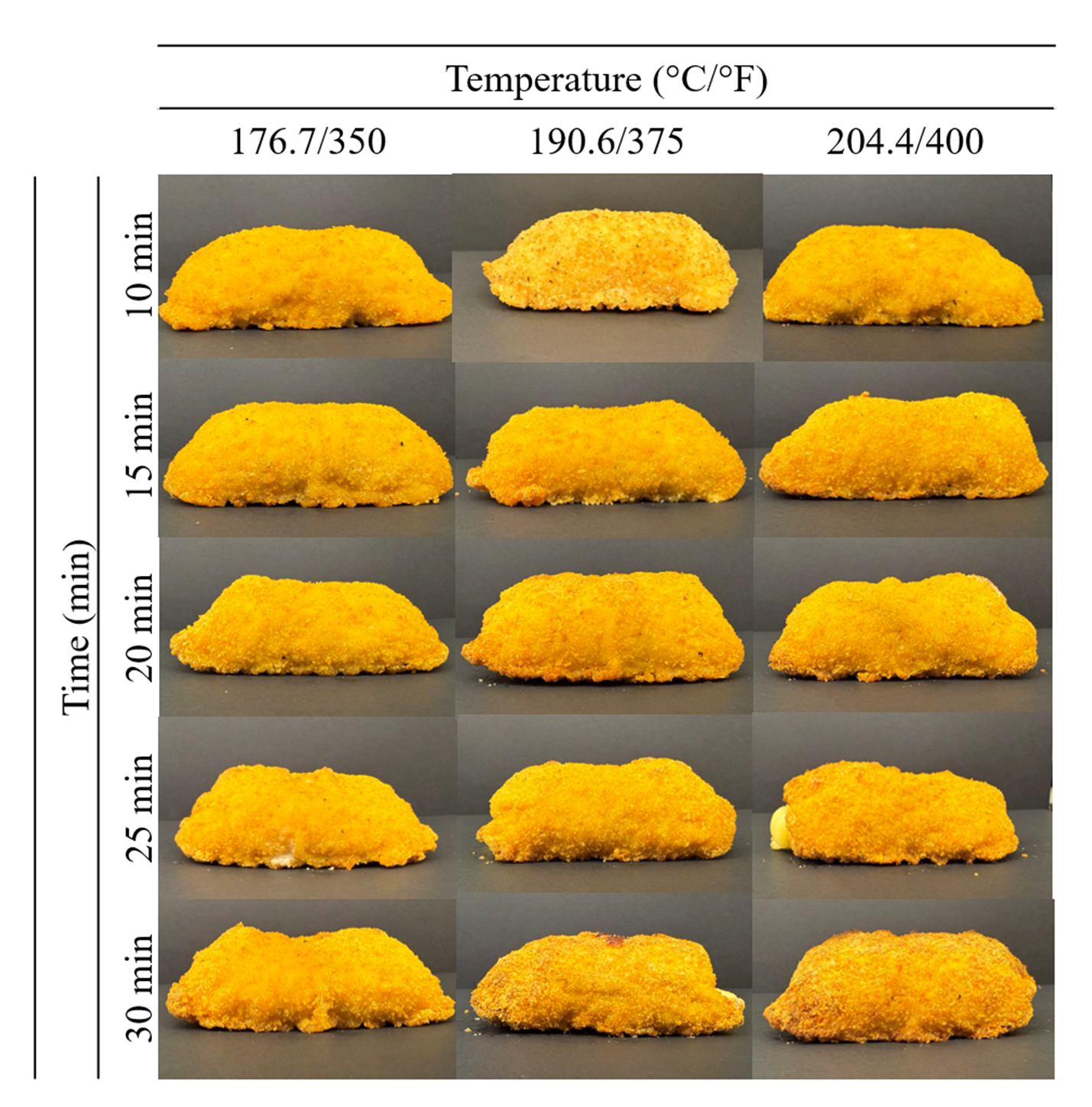
**

**Figure S2.** The air fryer temperatures, (A) basket-type at 176.7 °C (350 °F), (B) basket-type at 190.6 °C (375 °F), (C) basket-type at 204.4 °C (400 °F), (D) oven-type at 176.7 °C (350 °F), (E) oven-type at 190.6 °C (375 °F), (F) oven-type at 204.4 °C (400 °F)

**
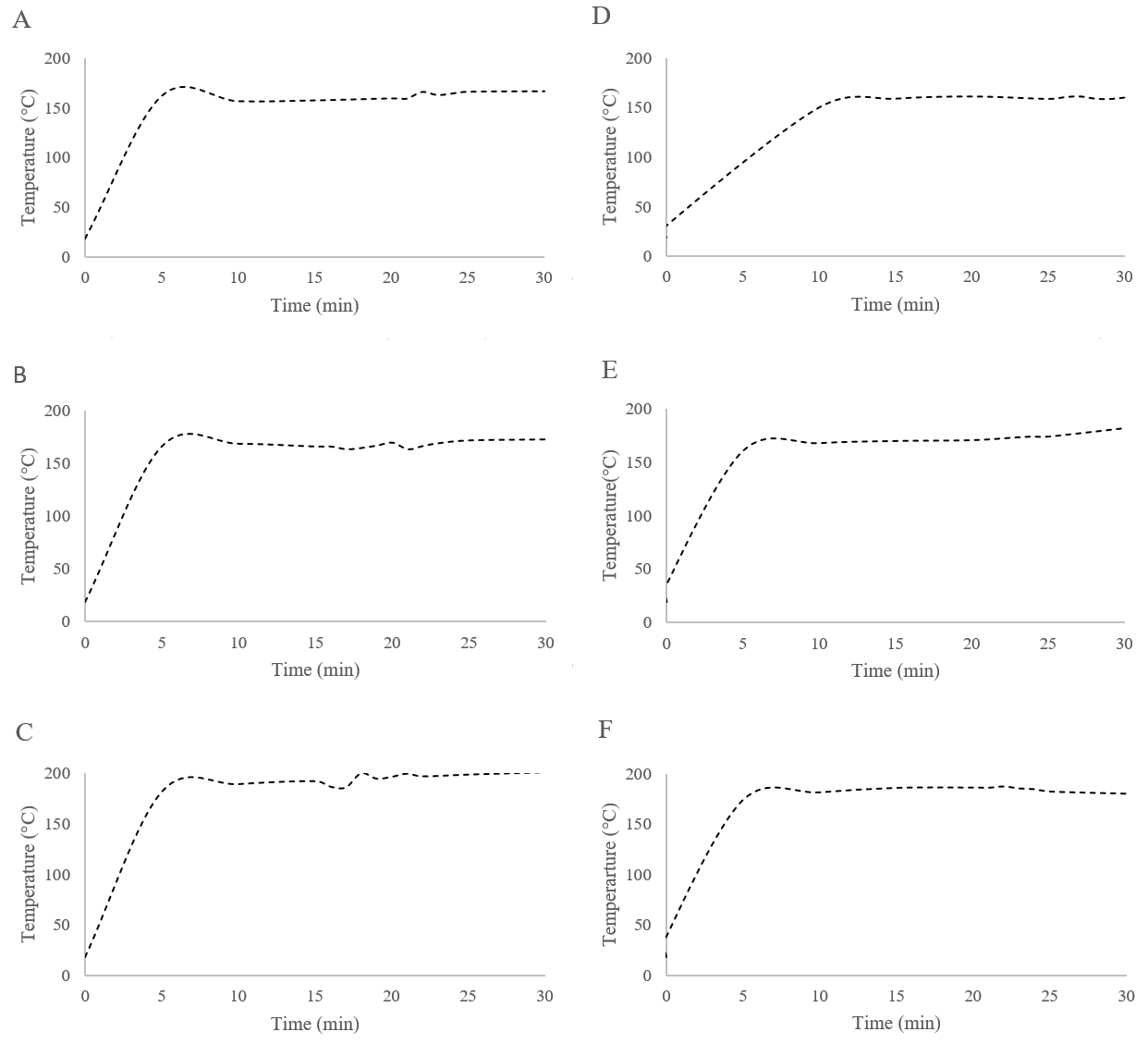
**

**Figure S3.** The shear curve for the MORS test of control sample
